# What's in a Word? On Weight Stigma and Terminology

**DOI:** 10.3389/fpsyg.2016.01527

**Published:** 2016-10-05

**Authors:** Angela Meadows, Sigrún Daníelsdóttir

**Affiliations:** ^1^School of Psychology, University of BirminghamBirmingham, UK; ^2^Division of Health Determinants, Directorate of HealthReykjavik, Iceland

**Keywords:** weight stigma, weight bias, anti-fat prejudice, obesity, terminology, language, social justice, fat activism

In 2015, the 3rd Annual International Weight Stigma Conference was held in Reykjavik, Iceland.^1^ One of the highly anticipated sessions of the 2-day event was a roundtable discussion on terminology used in weight stigma research and professional practice to describe higher-weight bodies and to identify best practice—how to engage in the conversation without being part of the problem. We tried to include a range of voices on the panel, including weight stigma researchers from health and social sciences, a bioethicist, a journal editor, a representative of an obesity organization, and a size-acceptance activist. At the end of the hour, the only thing that everybody agreed on was that there was no simple answer, other than to respect and honor the wishes of the person or people we were speaking to or about in any given situation.

Part of the problem is that the very act of labeling is a process of othering, one that creates a distinction between us and them; which raises the question: who is entitled to do the labeling and why, and in what conditions is such a distinction needed? For example, it is undoubtedly useful to define a group for research purposes, for example, so that the barriers and discrimination they face can be quantified and addressed. However, within the medical setting, the main reason to create a separate category for larger bodies is because they are to be treated differently than slimmer patients. Whether or not such differential treatment is perceived to be necessary reflects fundamentally divergent framings of higher-weight bodies. It is unlikely there can ever be agreement between people whose “solution” to body diversity is social justice and acceptance of this diversity, and those whose “solution” is elimination of the difference.

And yet, there has been a move in recent years, particularly among weight-focused research journals, to mandate the ubiquitous use of “person-first” language, such as “person with obesity,” rather than “obese person” (Kyle and Puhl, 2014; Wittert et al., 2015). Person-first language originated through disability advocacy (Blaska, 1993), and many organizations now recommend or obligate phrases such as “person with disability” in place of “disabled person.” Yet the term is far from universally accepted, particularly among the target population (Jernigan, 1993; Vaughan, 1993; Sinclair, 1999; Liebowitz, 2015). Given the current promotion of its use in the “obesity” field, it is worth looking a little more closely into how person-first language contributes to the ongoing and increasing stigmatization of heavier bodies.

The origin and intention of the phrase is superficially benevolent, suggesting that a person be considered wholistically and not defined by a particular (negative) characteristic. However, a number of new, and likely unintended, consequences arise from this approach. Hudak (2001 cited in Smith et al., 2007), distinguished between “benign” and “toxic” labeling, where the former is simply descriptive but the latter can lead to oppression and stigmatization. It would be considered absurd to describe a native of Germany, for example, as a “person with German-ness” because adjectives associated with nationality are descriptive and (usually) unvalenced. In contrast, the apparent need to separate a person from the characteristic in question implies an inherent adverse judgment. Second, the idea that we are all people but some of us are “burdened” with this millstone around our neck both denotes that only by fixing or removing this blight can we become like “everyone else,” and precludes that we can ever be “normal” in our current form (Titchkosky, 2001). Thus, far from returning our humanity to us or fostering our dignity, we are marked with a defect, the very definition of stigma proffered by Goffman in his seminal work on the nature of spoiled identity (Goffman, 1963).

What is more, person-first language is mired in the medicalization of body state. Since the American Medical Association controversially declared “obesity” a disease in 2013 (Frellick, 2013), in contravention to the recommendations of their own scientific committee (AMA Council on Science and Public Health, 2013), the result has not been that heavier people are treated more respectfully, or viewed by the medical profession in their complete personhood. Rather, anti-fat attitudes remain high among health professionals and specialists in the field (Flint and Reale, 2014; Puhl et al., 2014a,b; Tomiyama et al., 2015; Garcia et al., 2016), and the Endocrine Society even went so far as to release guidelines suggesting clinicians should treat the “obesity” before all else, prioritizing weight management over clinical effectiveness and tolerability in prescribing choices for conditions such as schizophrenia, epilepsy, depression, and HIV (Apovian et al., 2015; Tucker, 2015).

And yet, resolving to use the language preferred by the target group itself does not simplify the decision. While some obesity organizations that call for the use of person-first language claim to speak for all higher-weight people, this population is far from homogeneous, and individuals who do engage with such organizations will be a self-selecting group who are seeking a medical solution to something they consider inherently problematic. Indeed, a coalition of size-acceptance and fat rights groups have challenged the claim that these organizations speak for larger people as a whole, criticizing the top-down setting of the terminology agenda and the absence of grassroots input from social justice organizations that fight for fat people's interests (NAAFA, 2015).

In contrast, several studies have attempted to ascertain actual individual preferences. A number of these studies have used the Weight Preference Questionnaire (Dutton et al., 2010; Volger et al., 2012; Puhl et al., 2013), which asks individuals to rate their preference for 11 terms that a doctor could use to start a discussion about them being “at least 50 pounds over [their] recommended weight.” However, two factors limit the validity of this measure to identify language preferred by higher-weight people to describe their bodies. First, the questionnaire prompts participants *a priori* to think of weight as a problem. Secondly, the 11 terms used in the Weight Preference Questionnaire were chosen after consultation with patients in treatment-seeking settings (Wadden and Didie, 2003). Thus, neither the list of words generated, nor the scenario used in the exercise, is judgment-free. Other studies looking at terminology preference, while not necessarily recruiting treatment-seeking patients, have also been framed in terms of language to be used in a clinical setting to discuss “problem” weight (Eneli et al., 2007; Ward et al., 2009; Puhl et al., 2011; Knierim et al., 2015). All of the above studies reported similar findings—neutral terms such as “weight” or “BMI” were preferred, independent of participant age, gender, ethnicity, or BMI. Phrases including the words “problem,” “unhealthy,” or “excess” were preferred less. In all cases, “obesity” and “fat” were regarded as the least desirable. The implications of this for clinical practice are reasonably clear, but the generalizability of the findings to other contexts is debatable, and these may not be the words that would be chosen by heavier people outside of a weight-loss setting.

Support for this contention comes from a qualitative study of the lived experience of 76 Australian adults with a BMI greater than 30 (Thomas et al., 2008). The sample included a wide age range and most had been heavier for the majority of their lives. Almost all had experienced weight stigma at some point. While the participants were all unhappy with their weight, and felt responsible for changing it, 80% of them hated or disliked the words “obesity” and “morbidly obese,” and would rather be called “fat” or “overweight.” Thus, although the medical establishment positions “obesity” as a neutral term, higher-weight individuals do not seem to like it, and associate it with increased societal disapproval.

Importantly, Smith et al. (2007) noted that words such as “obese,” “overweight,” and “heavy” are often used interchangeably, assuming that their meaning is equivalent in the eyes of the researchers and among research participants. However, their research demonstrated that a fictional woman who described herself in a personal ad using either what the researchers labeled as positive (full-figured), negative (fat, obese, overweight), or objective (197 pounds) terms to describe her weight, was rated differently on friendliness, attractiveness, and intelligence based on which terms were used, and was always rated more positively when no weight descriptor was included in the ad. Interestingly, ratings of her level of fatness also varied significantly depending on the term used. Further, a series of studies by Brochu and Esses (2011) suggest that even though students assign similar body size silhouettes to people labeled as “fat” and “overweight,” they rated “fat” people significantly less favorably than “overweight” people using an attitude thermometer (and rated both less favorably than seven other social groups), attributed more negative characteristics to the “fat” person, and were less likely to recognize “fat” people as being targets of discrimination than “overweight” people. They found the effect was mediated via greater endorsement of negative weight-related stereotypes in a “fat” compared with an “overweight” target.

Yet, despite the word “fat” being almost universally considered pejorative within the wider community (Brochu and Esses, 2011; Trainer et al., 2015), it is the preferred term within the fat acceptance movement, whose reclamation of the word as a neutral descriptor aims to counter the negative stereotypes that have become associated with it, and normalize the existence of fat bodies (Saguy and Ward, 2011). Thus, identifying as “fat” becomes an act of empowerment and a marker of self-respect and unity. The same approach has been utilized by other human rights groups, such as the LBGTQ movement's embracing of terms that have historically been used to shame and marginalize them (Brontsema, 2004).

Ideally, it should be the target group itself that gets to decide on the label used to describe them. To date, however, research on the preferences of this group has been skewed toward treatment-seeking populations (Wadden and Didie, 2003; Dutton et al., 2010; Volger et al., 2012), and therefore the findings of such research cannot be regarded as representing a “consensus.” Weight-loss seeking populations differ from their non-treatment seeking peers in many respects, such as health status, self-acceptance, and empowerment (McKinley, 2004; Lewis et al., 2011; Blake et al., 2013; Cernelič-Bizjak and Jenko-Pražnikar, 2014). A very different picture would likely emerge from groups involved in size-acceptance activism, and yet their voices have generally not been included in efforts to engage with the target population. As an analogy, this would be similar to asking women, in the early days of the feminist movement, about their views on women's social status, without including participants with a feminist viewpoint. Many women at that time would have agreed with paternalistic views, such as that the woman's role was to stay home with the children and the man was the head of the household (Downing and Roush, 1985; Steuter, 1992). In fact, during important struggles in the women's rights movement, some groups of women actively fought *against* those demanding legal and civil rights (Ehrenreich, 1981; Graves, 2006). Thus, consensus within a socially marginalized group can neither be realistically expected nor made to serve as a prerequisite for moving toward social justice and equality. While the word “fat” may still be viewed negatively by many people, if prior human rights struggles are any indicator, it is likely to gain increased public acceptance as the fight for body equality evolves. We are currently at a moment in history where this fight has only just begun, and we are bound to witness considerable changes in the way we think about bodies, and acceptable terms for those bodies, in the years to come.

Ultimately, whether you describe somebody as “fat,” “overweight” “obese,” “big,” “heavy,” “voluptuous,” or simply “higher-weight,” these labels all reflect certain culturally constructed values. It behooves us to ask ourselves whether the words we use do indeed affirm the respect and human dignity of the target group, whether they place the group as equal to other social groups, and whether they promote or hamper the wellbeing and empowerment of that group. If not, we will only perpetuate the stigma we are claiming to abolish. As a first step, we suggest that best practice in research, publishing, and healthcare would be to use neutral terms, with “weight” and “higher weight” likely to be suitable in the majority of situations. We would also exhort journal editors to remove the insistence on person-first terminology that precludes more nuanced consideration of the implications of language use.

## Author contributions

AM and SD both contributed to the conception and writing of this paper, and final approval of the written version. AM and SD agree to be accountable for all aspects of the work in ensuring that questions related to the accuracy or integrity of any part of the work are appropriately investigated and resolved.

### Conflict of interest statement

The authors declare that the research was conducted in the absence of any commercial or financial relationships that could be construed as a potential conflict of interest.

## Footnotes

^1^Stigmaconference.com.
